# *Achromobacter* spp. genetic adaptation in cystic fibrosis

**DOI:** 10.1099/mgen.0.000582

**Published:** 2021-07-07

**Authors:** Migle Gabrielaite, Finn C. Nielsen, Helle K. Johansen, Rasmus L. Marvig

**Affiliations:** ^1^​ Center for Genomic Medicine, Rigshospitalet, Copenhagen, Denmark; ^2^​ Department of Clinical Microbiology, Rigshospitalet, Copenhagen, Denmark; ^3^​ Department of Clinical Medicine, Faculty of Health and Medical Sciences, University of Copenhagen, Copenhagen, Denmark

**Keywords:** within-host evolution, host-pathogen interaction, microbial genomics, genomic adaptation, *Achromobacter*, cystic fibrosis airway infection, GWAS

## Abstract

*Achromobacter* spp. are emerging pathogens in patients with cystic fibrosis (CF) and *Achromobacter* spp. caused infections are associated with more severe disease outcomes and high intrinsic antibiotic resistance. While conventional CF pathogens are studied extensively, little is known about the genetic determinants leading to antibiotic resistance and the genetic adaptation in *Achromobacter* spp. infections. Here, we analysed 101 *Achromobacter* spp. genomes from 51 patients with CF isolated during the course of up to 20 years of infection to identify within-host adaptation, mutational signatures and genetic variation associated with increased antibiotic resistance. We found that the same regulatory and inorganic ion transport genes were frequently mutated in persisting clone types within and between *Achromobacter* species, indicating convergent genetic adaptation. Genome-wide association study of six antibiotic resistance phenotypes revealed the enrichment of associated genes involved in inorganic ion transport, transcription gene enrichment in β-lactams, and energy production and translation gene enrichment in the trimethoprim/sulfonamide group. Overall, we provide insights into the pathogenomics of *Achromobacter* spp. infections in patients with CF airways. Since emerging pathogens are increasingly recognized as an important healthcare issue, our findings on evolution of antibiotic resistance and genetic adaptation can facilitate better understanding of disease progression and how mutational changes have implications for patients with CF.

## Data Summary

*Achromobacter* spp. whole-genome sequencing data is available at European Nucleotide Archive under study accession number PRJEB39108.

**Impact Statement**

*Achromobacter* species are increasingly detected in patients with cystic fibrosis (CF) in which they can cause chronic airway infections. However, the knowledge about how *Achromobacter* spp. genetically adapt to the human airway environment is lacking. To address these questions, we analysed 101 genomes of *Achromobacter* spp. from 51 Danish patients to investigate within-host genetic changes of *Achromobacter* spp. over up to 20 years of airway infections. We identified convergent evolution patterns of regulatory and inorganic ion transport genes. We additionally found that genes involved in inorganic ion transport, transcription, and energy production and translation were associated with antibiotic resistance phenotypes. Altogether, our analysis revealed the principal genomic adaptation and antibiotic resistance development patterns of *Achromobacter* spp. during the infection in patients with CF airway. The gained knowledge from our work helps to better understand how changes in the *Achromobacter* spp. genomes impact the disease progression in patients with CF and facilitates the identification of the improved treatment strategies of CF airway infections.

## Introduction

*Achromobacter* spp. are emerging pathogens causing chronic bacterial infections in patients with CF [1–4]; however, it is still unclear to what extent *Achromobacter* spp. infections impact morbidity and mortality in these patients [3–5]. Analysis of pathogen genomes, i.e. pathogenomics, have shown that within-host pathogen genetic adaptation plays a role in these infections [6, 7]. While conventional CF pathogens, e.g. *Pseudomonas aeruginosa* and *Staphylococcus aureus*, are studied extensively, little is known about the extent within- and between-patient genetic adaptation has in *Achromobacter* spp. infections, particularly *A. ruhlandii, A. insuavis* and *A. xylosoxidans* as they were shown by Gade *et al.* to be the main cause of chronic, long-term infections in patients with CF infected with *Achromobacter* spp. [1, 4, 8–10] Furthermore, genetic features, when paired with phenotypic observations, could be used for successful resistance profile predictions as conventional methods are both time consuming and occasionally do not reflect the *in vivo* susceptibility profiles [11–13]. Knowledge of within-host *Achromobacter* spp. adaptation and genetic factors leading to antibiotic resistance development are key for urgently needed new treatment strategy development and pathogen elimination.

Here, we analysed 101 previously whole-genome-sequenced (WGS) *Achromobacter* spp. isolates from 51 patients to investigate the genetic relatedness and within-host genetic changes of *Achromobacter* spp. over the course of up to 20 years of infections. First, we aimed to identify the main gene content differences between and within *Achromobacter* spp. isolates. Second, we aimed to identify pace and patterns in genetic changes, and compare it to the other pathogenic bacteria in CF. Finally, we attempted to define the most significant associations between *Achromobacter* spp. genetic features and antibiotic resistance phenotypes. Ultimately, this work on the main *Achromobacter* spp. genomic changes acquired during infections in patients with CF, leads to the possibility of genomic-based disease progression prediction, and improved strategies to track and treat persistent airway infections.

## Methods

### Bacterial isolates

Our analysis included 101 clinical isolates of *Achromobacter* spp. that were defined in detail previously by Gabrielaite *et al*. [14]. The isolates were sampled from 51 patients with CF attending the Copenhagen Cystic Fibrosis Center at Rigshospitalet, Copenhagen, Denmark. Over the timespan of 0–20 years (median 6.5 years), 64 isolates from 25 patients were longitudinally collected (median 2 isolates) and 37 isolates from 29 patients were single isolates. Overall, 29, 18 and 52 isolates belonged to *A. ruhlandii*, *A. insuavis* and *A. xylosoxidans*, respectively. Furthermore, single isolates belonging to *A. aegrifaciens* and a new genogroup were sequenced.

### Bacterial genome sequencing and definition of clone type

Genomic DNA was prepared from *Achromobacter* spp. isolates using a DNeasy Blood and Tissue kit (Qiagen) and sequenced on an Illumina MiSeq platform, generating 250 base paired-end reads. On average, 1124551 reads (range of 350677–2118817) for each of the genomic libraries were obtained. Clone types were defined by Pactyper [15] using the default parameters and species’ core genome defined by GenAPI [16]. Lineage was defined as all isolates belonging to the same species and the same clone type.

### Bacterial genome assembly

Sequence reads from each isolate were corrected and assembled by SPAdes 3.10.1 [17] using default parameters and k-mer sizes ranging from 21 to 127. Assembled contigs were joined to 216 scaffolds on average (92–506).

### Average nucleotide identity calculation

Wrongly annotated public *Achromobacter* species from RefSeq database [18] were identified by calculating ANI with fastANI 1.11 [19] using 95 % threshold.

### Aggregated pan-genome generation, characterization and visualization

Aggregated pan-genome was created by clustering all pan-genomes from longitudinally collected lineages and *de novo* assemblies from single-isolate lineages with GenAPI [16]. Every gene in the aggregated pan-genome was then aligned back to the individual pan-genomes/*de novo* assemblies to determine if the gene is (1) non-present in the lineage (2), present and variable within the lineage or (3) present and non-variable. A matrix for an aggregated pan-genome was generated for 26 longitudinal lineages and 35 single-isolate lineages, and visualized using R [20] with a pheatmap library [21].

### Bacterial genome alignment and variant calling

Alignments, variant calling and pairwise SNP distance identification for *Achromobacter* spp. isolates were performed using reference genomes [GCF_001051055.1 for *A. ruhlandii* (AX01 group), GCF_001558755.2 for *A. insuavis* (AX02 group) and GCF_001457475.1 for *A. xylosoxidans* (AX03 group)] with BacDist [22] workflow that is based on variant calling with Snippy [23]. Sequence alignments on average included 84 % (81.72–89.58 %) of the raw sequencing reads for *A. ruhlandii*, 87 % (75.45–92.57 %) for *A. insuavis* and 86 % (75.95–93.61 %) for *A. xylosoxidans*. Low-quality variants (<tenfold coverage or supported by <50 % of mapped reads) or variants shared among all isolates were discarded by BacDist (see Gabrielaite *et al*. [14] for detailed tool description).

### Substitution rate estimation

The nucleotide substitution rate [24] estimation was performed for each lineage containing three or more isolates sampled at different timepoints (ten lineages in total) by using beast 2.6.1 [25]. Sequence alignments from BacDist were used as input with the following parameters: [1] sequences were annotated with the sampling date (‘dated tips’) [2], HKY substitution model with strict clock parameters [3], gamma prior for clock rate [4], prior for population size: 1/X [5], tree prior: coalescent constant population. MCMC was run for 50 000 000 iterations. Convergence was checked by inspecting an effective sample size and parameter value traces in the Tracer 1.7.1 software [26]. Multiple tests for each sample were performed to ensure reproducibility and convergence. The obtained clock rate (per site per year) was multiplied by the alignment size to obtain a substitution rate per genome per year.

### Virulence and antibiotic resistance gene identification

Orthologues of resistance and virulence genes in 61 *Achrmobacter* spp. lineages were identified with Abricate [27] using VFDB (containing 2597 genes; retrieved: 21 September 2020) [28] for virulence genes and Resfinder 4 (containing 3122 genes; retrieved: 21 September 2020) [29] for resistance genes. Gene orthologue was considered present in the corresponding database if the alignment made up minimum 50 % of the gene length and its identity was minimum 75 %.

### Frequently mutated gene definition

Most frequently mutated genes were defined as the top 1 % of all mutated genes for the species. If there were more genes mutated with the same frequency as the 1 % most frequently mutated genes, these genes were also included in the final analysis. The identified most mutated genes were annotated by EGGNOG-mapper 2.0.1 [30] using DIAMOND and EGGNOG’s bacterial database.

*P. aeruginosa* orthologues were identified by performing clustering with CD-HIT [31] using word size of 3 and 50% identity thresholds. Joint *Achromobacter* spp. most frequently mutated genes were identified by clustering with CD-HIT [31] with word size of 3 and 80% identity threshold.

### Genome-wide association study with antibiotic resistance phenotypes

dbgwas 0.5.4 [32] software was used for bacterial genome-wide association analysis using ten [Amoxicillin-Clavulanate (AMC), Ceftazidime (CAZ), Chloramphenicol (CHL), Colistin (CST), Imipenem (IPM), Meropenem (MEM), Piperacillin-Tazobactam (TZP), Sulfamethizole (SMZ), Tigecycline (TGC) and Trimethoprim-Sulfamethoxazole (SXT)] different antibiotic resistance phenotypes and *de novo* assembled scaffolds of 92 isolates for which the antibiotic susceptibility profiles were available (Gabrielaite *et al*. [14] for detailed information on isolate susceptibility). Core genome SNP-based phylogenetic tree was used to correct for population structure while all available annotations of *Achromobacter* spp. genes from UniProt database [33] were used for unitig annotation (271851 genes; retrieved: 19 April 2020). Ten of the most significant unitigs for each antibiotic test were used for further analysis as the tool authors advise against using a *P*-value threshold when testing several phenotypes. [32] *Achromobacter* spp. gene annotations were identified by clustering significant unitig gene sequences with CD-HIT [31] with word size of 3 and 80% identity threshold. Enrichment of COGannotated genes was estimated by comparing the fraction of the associated genes with the fraction in the *Achromobacter* spp. reference genomes for the five most frequently associated gene groups where more than one gene was present in a group.

## Results

### *Achromobacter* spp. dataset and incorrect annotations of public genomes

**Fig. 1.**
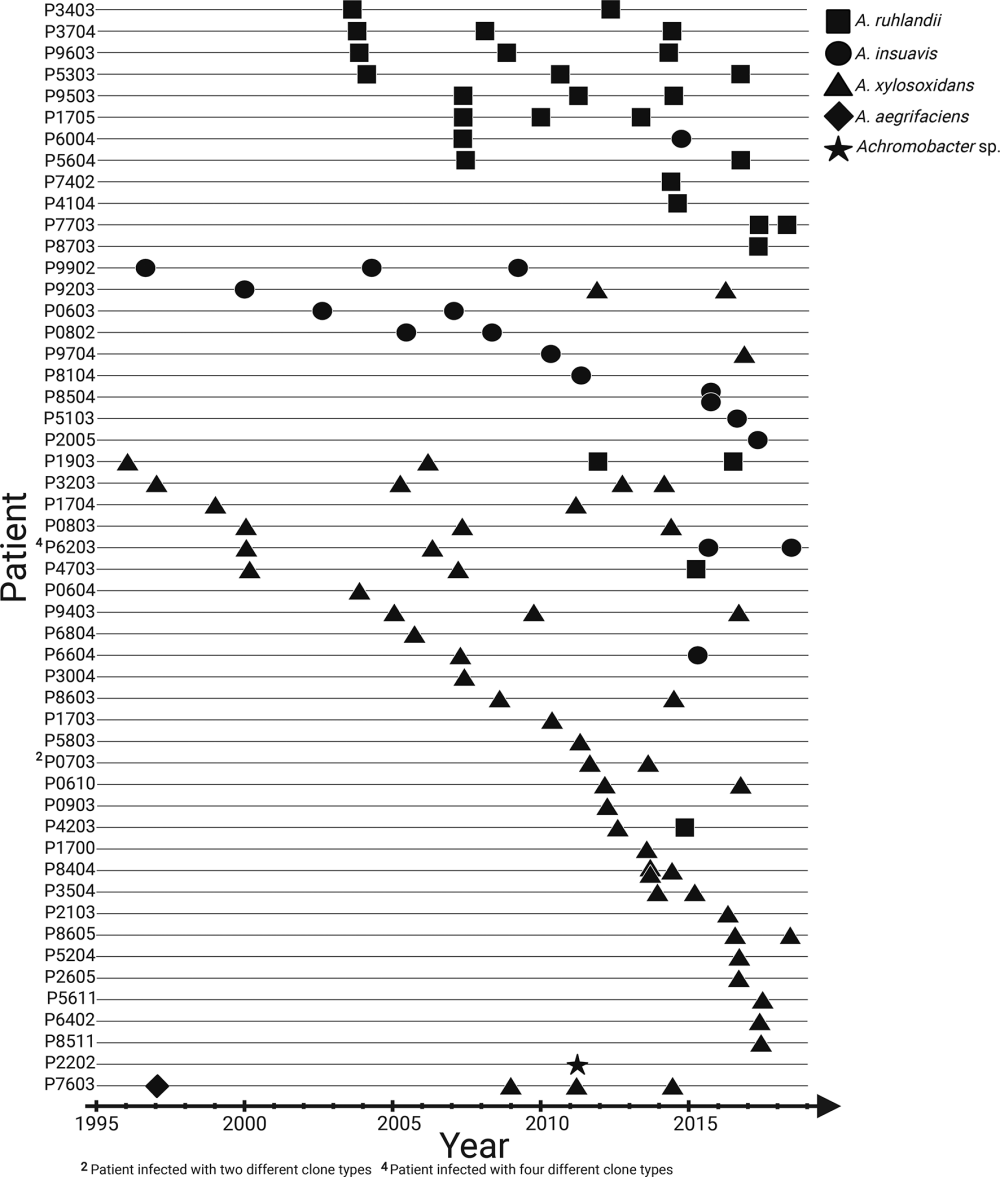
Overview of 101 longitudinally collected *Achromobacter* spp. isolates from patients with CF.

The genomes of 101 *Achromobacter* spp. isolates from the airways of 51 patients with CF attending the Copenhagen Cystic Fibrosis Center at Rigshospitalet were sequenced to follow the within-host evolution and genetic adaptation of the lineages over the initial 0–20 years of infection (Fig. 1). All isolates were previously defined as belonging to five different species: *A. ruhlandii* (*N*=29), *A. insuavis* (*N*=18), *A. xylosoxidans* (*N*=52), *A. aegrifaciens* (*N*=1) and a new genogroup (*N*=1). The latter two species were excluded from further analysis as the species contained only a single isolate. The remaining 99 *Achromobacter* spp. genomes were grouped to 61 lineages, which were defined as all isolates from one patient belonging to the same species and clone type (Table S1, available in the online version of this article).

**Fig. 2.**
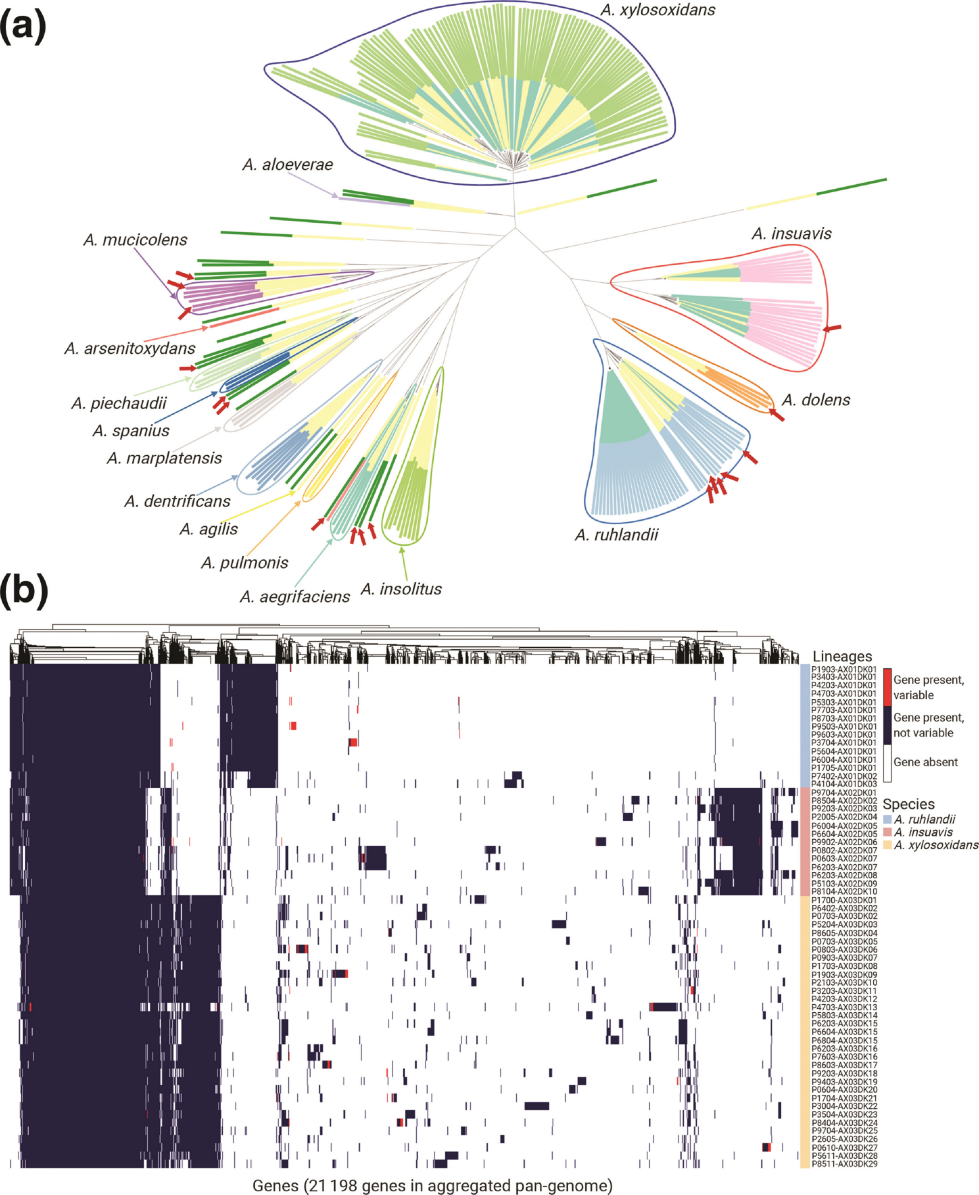
*Achromobacter* spp. genetic differences. (a) Phylogenetic tree based on core genome SNPs of 101 *Achromobacter* spp. isolates from patients with CF (inner layer of colours: turquoise) and 141 *Achromobacter* spp. isolates from RefSeq database (inner layer of colours: yellow). Outer layer of colours corresponds to species annotation with suggested corrections; red arrows mark supposedly incorrect species annotation in RefSeq isolates. The phylogenetic tree can be accessed on Microreact webserver [66]. (b) The aggregated pan-genome of 61 *Achromobacter* spp. lineages containing gene presence, absence, and gene variability (i.e. gene is present in some isolates while absent in other isolates within the lineage) information.

We first performed phylogenetic analysis for all *Achromobacter* spp. genomes available in the RefSeq database (141 samples, Table S2) together with our clinical *Achromobacter* spp. genomes. Our sequenced genomes were widely distributed across the genetic variability observed within the *A. insuavis* and *A. xylosoxidans* group; however, *A. ruhlandii* isolates, of which the majority (27/29) belonged to Danish epidemic strain (DES), reflected little genetic variability of *A. ruhlandii* species. Furthermore, our phylogenetic and average nucleotide identity (ANI) analysis revealed that *Achromobacter* spp. annotations are inconsistent among the RefSeq genomes and require corrections to improve species designation (Fig. 2a, suggested corrections in Table S2).

### Aggregated pan-genome

Aggregated pan-genome was constructed from pan-genomes of each of the 61 lineages (35 of which were single-isolate lineages; Table S1). This approach allowed us to account for the nature of the dataset where multiple clonal isolates from the same patient were available. The aggregated *Achromobacter* spp. pan-genome consisted of 21198 genes: 2887 core genes, 18311 accessory genes of which 6917 genes were unique to a single lineage.

The aggregated pan-genome (Fig. 2b) contained *Achromobacter* species-specific genes (649 for *A. ruhlandii*, 648 for *A. insuavis*, and 494 for *A. xylosoxidans*) present in all isolates of the respective species but not in the isolates from other species. Pan-genomes for each *Achromobacter* species were defined by using all bacterial isolates available [18–52] for the species. The size of the species' pan-genomes contained 7070–14833 genes, of which 4225–5130 were core genes, 1940–10608 accessory genes and 976–3162 isolate-unique genes (Table 1).

**Table 1.** Pan-genome size, number of core, accessory and unique genes for isolates from each *Achromobacter* species

Species	No. of bacterial isolates	Pan-genome size	Core genes	Accessory genes	Unique genes
*** A. ruhlandii ***	29	7070	5130	1940	976
*** A. insuavis ***	18	9900	4799	5101	1124
*** A. xylosoxidans ***	52	14833	4225	10608	3162

### *Achromobacter* spp. substitution rates

Within-patient bacterial substitution rate was estimated for lineages where three or more *Achromobacter* sp. isolates from different timepoints were available (five lineages for *A. ruhlandii*, four for *A. xylosoxidans* and one for *A. insuavis*). The estimated substitution rates for *A. ruhlandii* (DES isolates which are known hypermutators [14, 34]) were on average 4.18·10^−6^ (2.71·10^−6^–5.39·10^−6^) SNPs/year/site, 8.77·10^−7^ (6.17·10^−7^–1.13·10^−6^) SNPs/year/site for *A. xylosoxidans* (0332-AX03DK11 hypermutator lineage [14] with the mutation rate of 2.37·10^−6^ SNPs/year/site was excluded), and 1.61·10^−7^ SNPs/year/site for *A. insuavis*. These substitution rates correspond to an average of 21.5, 2.19 and 0.79 SNPs/year/genome for *A. ruhlandii*, *A. xylosoxidans* and *A. insuavis*, respectively (Fig. S1).

### Virulence and antibiotic resistance genes carried by *Achromobacter* spp. genomes

**Fig. 3.**
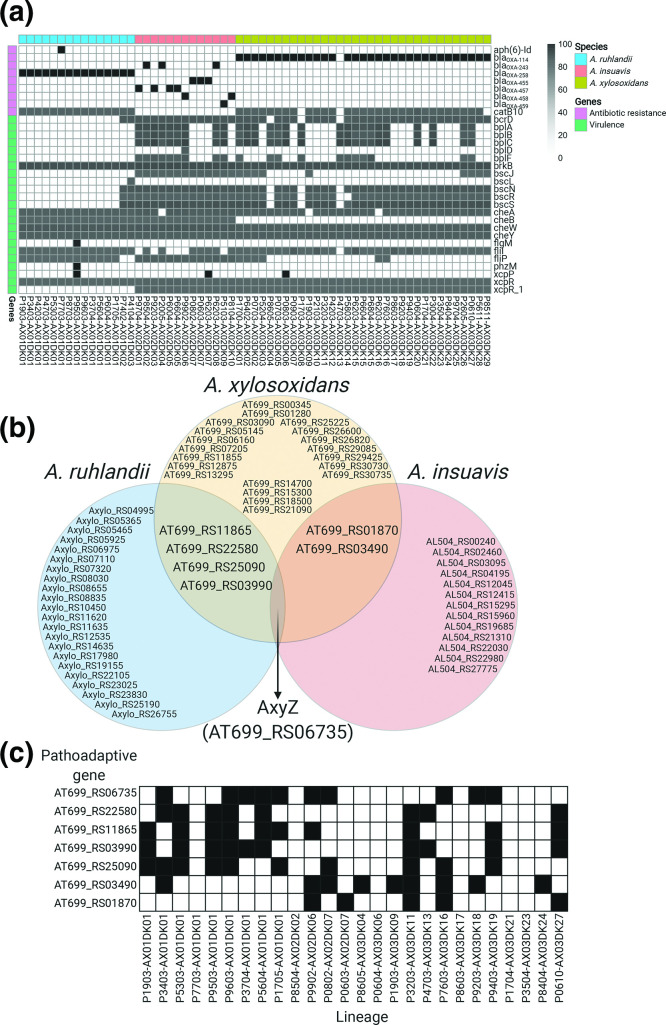
Overview of genetic determinants related to virulence and antibiotic resistance, and pathoadaptive genes. (a) Antibiotic resistance and virulence gene orthologue distribution among lineages. (b) Venn diagram of the most frequently mutated genes and their overlap between the three *Achromobacter* species. (c) Candidate pathoadaptive gene mutation distribution by lineage.

From *de novo* assembled genomes of the 99 *Achromobacter* spp. isolates we identified virulence (VFDB database) and antibiotic resistance (Resfinder database) gene orthologues. On average, bacterial isolates carried 2 (0–3) antibiotic resistance gene orthologues and 13 [8–18] virulence gene orthologues. The most frequently carried antibiotic resistance gene was the orthologue of *catB10,* which codes for chloramphenicol acetyltransferase. Interestingly, the gene was carried by all *A. ruhlandii* and nearly all (30/33) *A. xylosoxidans* lineages but none of the *A. insuavis* lineages (Fig. 3a). Furthermore, OXA-type class D β-lactamase *bla*_OXA-258_ was observed in all *A. ruhlandii* isolates and *bla*_OXA-114_ – in all but one (P7034-AX03DK13) *A. xylosoxidans* lineages. All 13 *A. insuavis* isolates carried one of the following *bla*_OXA_ genes: *bla*_OXA-243_, *bla*_OXA-455_, *bla*_OXA-457_, *bla*_OXA-458_ or *bla*_OXA-459_. All *bla*_OXA_ genes carried by *A. insuavis* had ≥92 % nucleotide identity. The latest isolate of DES (P7703-AX01DK01) appears to have acquired a new aph [6]-Id antibiotic resistance gene orthologue encoding for aminoglycoside resistance [35].

The median number of virulence genes in *A. ruhlandii* DES genomes was markedly lower (*N*=8) than other *Achromobacter* spp. isolates (*N*=14; *P*-value=5.98·10^−8^; Wilcoxon signed-rank test) (Fig. 3a). The majority of virulence gene orthologues belonged to the secretion system (*N*=9), motility (*N*=7), and endotoxin (*N*=5) gene orthologs [28].

### Genetic adaptation: Mutations of the same genes across lineages

**Fig. 4.**
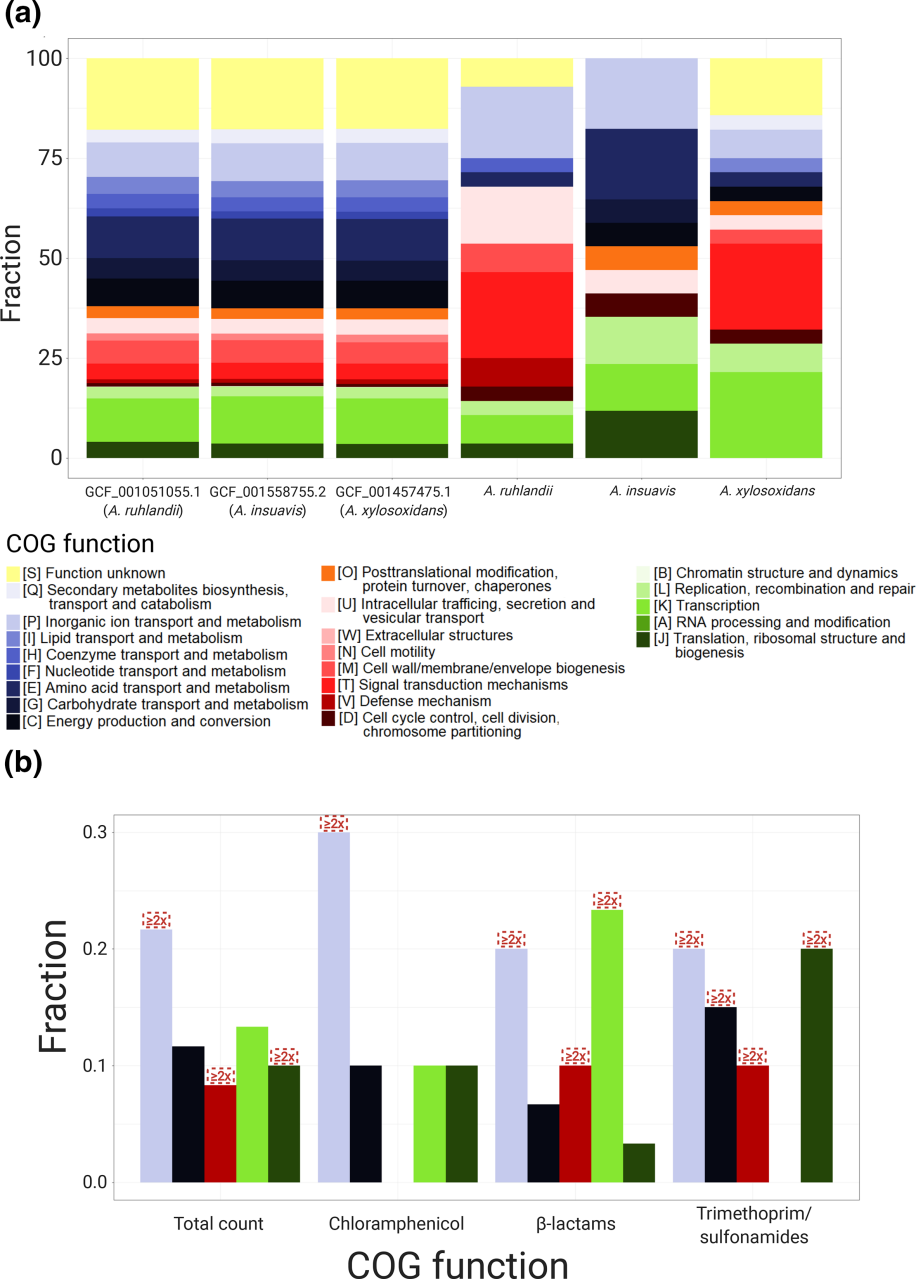
COG annotations of (a) three *Achromobacter* spp. reference genomes and most frequently mutated genes, and (b) frequently associated with antibiotic resistance unitigs. Red boxes mark COGs, which are ≥2x enriched in GWAS compared to reference genomes.

To explore within-host genetic adaptation in *Achromobacter* spp., we first identified the genes, which were most frequently mutated within each species. Genes were defined as frequently mutated if they were among the 1 % most commonly mutated genes within species. If more than 1 % of the genes were mutated with the same frequency, those genes were also included in the analysis. A total of 27, 16 and 28 genes were identified as most frequently mutated for *A. ruhlandii*, *A. insuavis* and *A. xylosoxidans*, respectively (Fig. 3b). The clusters of orthologous groups (COG) functional annotations were performed for all species (Fig. 4a, Table S3 for detailed information) with the highest mutation frequency in genes coding for signal transduction (COG T); inorganic ion transport and metabolism (COG P); replication, recombination and repair (COG L); intracellular trafficking, secretion and vesicular transport (COG U); and transcription (COG K).

Although knowledge is lacking about many *Achromobacter* spp. gene functions, bacterial sequence similarity analysis allowed us to identify possible antibiotic resistance and virulence-related genes among the most frequently mutated genes in *Achromobacter* spp. After manual literature search ten, four and four genes were defined as related to antibiotic resistance in *A. ruhlandii*, *A. insuavis* and *A. xylosoxidans*, respectively, whereas eight, two and seven genes were defined as virulence-related genes (Table S3).

Orthologue search of a previously defined list of 52 CF-associated pathoadaptive *P. aeruginosa* genes revealed three orthologues among the most frequently mutated *Achromobacter* genes: *mexZ* (WP_006389199.1), *mexB* (WP_024068614.1) and *gyrA* (WP_049072335.1).

Seven genes were defined as candidate pathoadaptive genes in several *Achromobacter* species. One gene was frequently mutated in all three species (10 out of 26 lineages) and six genes were observed as most frequently mutated in two of the *Achromobacter* species (Fig. 3c, Table 2). Six lineages did not acquire a single mutation in any of the seven pathoadaptive genes; however, 13 lineages acquired mutations in three or more genes (Fig. 3c). Lineages, which acquired mutations in three or more pathoadaptive genes, acquired markedly less non-synonymous mutations than lineages with less mutated pathoadaptive genes (*pP*-value=4.99·10^−2^; Fisher’s exact test). Furthermore, lineages only acquired non-synonymous mutations in the seven candidate pathoadaptive genes (Table S4).

**Table 2.** Seven most frequently mutated genes and their function

RefSeq ID (Locus tag)	No. of species	No. of lineages	Product	Function
WP_006389199.1 (AT699_RS06735)	3	10	DNA-binding transcriptional regulator AxyZ	Antibiotic resistance
WP_006227290.1 (AT699_RS01870)	2	5	NAD(P)/FAD-dependent oxidoreductase	Metabolic pathways
WP_013391523.1 (AT699_RS03490)	2	10	Penicillin-binding protein 2	Antibiotic resistance
WP_006392856.1 (AT699_RS03490)	2	9	ABC transporter substrate-binding protein	Transport
WP_006394390.1 (AT699_RS11865)	2	9	multidrug efflux RND transporter permease	Antibiotic resistance
WP_006387572.1 (AT699_RS22580)	2	8	Signal transduction histidine kinase	Two-component signalling
WP_006221301.1 (AT699_RS25090)	2	10	TonB-dependent hemin, ferrichrome receptor	Transport

The only pathoadaptive gene, which was frequently mutated among all three species – *axyZ –* encodes TetR family transcriptional regulator of the RND-type efflux system and is associated with innate *Achromobacter* spp. antibiotic resistance [36, 37]. Furthermore, we observed higher overall antibiotic tolerance by isolates, which acquired mutations in the *axyZ* gene with highest antibiotic resistance increase against piperacillin/tazobactam (*P*-value=3.28·10^−2^; Fisher’s exact test) and meropenem (*P*-value=2.87·10^−3^; Fisher’s exact test).

The ratio of non-synonymous to synonymous substitutions (d*N*/d*S*) was significantly different between the 1 % most frequently mutated genes and non-frequently mutated genes in *Achromobacter* spp. (d*N*/d*S*_99 %_=1.21 vs d*N*/d*S*_1 %_=2.10; Fisher’s exact test; *P*=3.4·10^−4^, respectively).

Finally, we investigated gene loss and acquisition patterns in 26 longitudinally collected *Achromobacter* spp. lineages. We observed genes to be two times more often lost than acquired, and lost/acquired in groups rather than individually; however, no convergent evolution patterns in *Achromobacter* spp. gene loss/acquisition were identified (detailed analysis in Text S1).

### Genome-wide association between *Achromobacter* spp. genotypes and antibiotic resistance

To test for associations between bacterial genetics and antibiotic resistance phenotypes we performed unitig-based DBGWAS analysis. Out of 21 antibiotics where the resistance was phenotypically tested, only ten antibiotics had both susceptible and resistant isolates from all three *Achromobacter* species. Accordingly, we performed the association analysis for these ten antibiotics (see Methods for detailed information). Unitigs passed a 5 % FDR corrected q-value threshold only for CHL, IPM, MEM, TZP, SMZ and SXT. Ten of the most significant unitigs were used for the six remainder association test analysis, resulting in 60 genes (50 unique genes) significantly associated with antibiotic resistance phenotypes (Fig. 4b, Table S6). The most abundant group of associated unitigs belonged to inorganic ion transport (*N*=13; 2.4× enriched; COG P) genes. The other four most abundantly associated genes belonged to transcription (*N*=8; COG K); energy production and conversion (*N*=7; COG C); translation, ribosomal structure and biogenesis (*N*=6; COG J), and defence mechanism (*N*=5; COG V) groups. Furthermore, transcription genes were enriched in the β-lactam (IPM, MEM and TZP) antibiotic group (*N*=7; 2.1× enriched) while translation, ribosomal structure and biogenesis genes were 5.4× enriched in trimethoprim/sulfonamides (SMZ and SXT) group (*N*=4). Defence mechanism genes were 9.2 and 9.0× enriched in β-lactam and trimethoprim/sulfonamide groups, respectively while energy production and conversion genes were 2.9× enriched in a trimethoprim/sulfonamide group.

Of nine antibiotic resistance genes from the ResFinder four database which were present in the aggregated pan-genome, none were associated with antibiotic resistance phenotypes in the GWAS analysis. Furthermore, core and accessory genes were equally associated with antibiotic resistance phenotypes (Table S6).

## Discussion

*Achromobacter* species are emerging pathogens causing chronic respiratory tract airway infections in patients with CF; however, the genetic epidemiology of these infections is little understood. We sequenced and analysed 101 genomes of *Achromobacter* spp. isolates from 51 patients with CF, which is the largest longitudinally collected *Achromobacter* spp. genome dataset available to date. This allowed us to investigate the population genomics and within-host adaptation, including genome-wide association analysis with antibiotic resistance phenotypes.

Phylogenetic analysis of our dataset with 141 publicly available *Achromobacter* spp. genomes from the RefSeq database revealed that our dataset well represented the genetic diversity of *A. xylosoxidans* and *A. insuavis* species. However, because of DES overrepresentation, *A. ruhlandii* isolates did not reflect the species genetic diversity. Furthermore, ANI together with core-genome-based phylogenetic analysis revealed that more than 10 % of publicly available *Achromobacter* spp. genomes are supposedly misannotated in the RefSeq database, which we anticipated to unravel to ease future research on *Achromobacter* spp. (Table S2).

From the aggregated pan-genome analysis we showed that core genome sizes were comparable between *A. ruhlandii*, *A. insuavis* and *A. xylosoxidans* which are similar to core genome size identified previously by Li *et al*. [38]. Nevertheless, the number of accessory and unique genes in species’ pan-genomes varied greatly as a result of the different number of independent genomes for each species. *A. ruhlandii* isolates mostly belonged to DES, which is spread through patient-to-patient transmission [14, 34], therefore, have lower pan-genome plasticity than *A. xylosoxidans* or *A. insuavis*.

Unlike many bacterial species, substitution rates for *Achromobacter* spp. are not known [39]. Our *A. ruhlandii* dataset only consisted of hypermutator DES lineages, which led to a high substitution rate estimate, and our substitution rate estimate for *A. insuavis* was based on only one longitudinally sampled lineage. *A. xylosoxidans* (8.77·10^−7^ SNPs/year per site) substitution rate is comparable to other Gram-negative bacterial species: *P. aeruginosa* (4.0·10^−7^ SNPs/year per site) [40], *Shigella sonnei* (6.0·10^−7^ SNPs/year per site) [41], *Echerichia coli* (2.26·10^−7^ SNPs/year per site) [42]. The substitution rate is substantially lower than *S. aureus* (1.87·10^−6^ SNPs/year per site) [43] or *Klebsiella pneumoniae* (1.9·10^−6^ SNPs/year per site) [44]. These findings suggest that *A. xylosoxidans* evolutionary rates are similar to other Gram-negative bacteria and the same models predicting evolutionary dynamics could be used [39]. Furthermore, the calculated substitution rates could be employed in future studies to estimate the date of possible lineage divergence or the occurrence of patient-to-patient transmission.

While it was previously suggested to use *bla*_OXA_ genes for *Achromobacter* species typing [45–47], we showed that in some cases such strategy would not be sufficient for species identification as isolates can carry none of the *bla*_OXA_ genes. Furthermore, we identified that *A. insuavis* can carry one out of several highly similar *bla*_OXA_ genes, which might further complicate such species typing approach. We also show that none of the *A. insuavis* isolates carried *catB10* chloramphenicol resistance gene orthologue; however, these identified resistance genes alone were not sufficient to explain the differences in antibiotic resistance phenotype between lineages and species.

Moreover, virulence gene orthologue analysis revealed that *Achromobacter* spp. carry several virulence factors with markedly less virulence genes in *A. ruhlandii* DES strain, which further supports the adaptive trade-off evolution hypothesis that virulence genes are not required or are selected against in chronic infections [39]. However, several virulence gene orthologues coding for host cell invasion (*cheW* and *cheY*) and facilitating evasion of the host immune response (*brkB* [48]) were observed in all lineages. Furthermore, our findings of secretion system, in particular type III secretion system, gene orthologues as the most prevalent virulence genes in *Achromobacter* spp. are in line with findings by Jeukens *et al*. and Li *et al*. [38, 49].

Among the candidate pathoadaptive genes (frequently mutated genes), we identified multiple antibiotic resistance genes, which were markedly more frequently mutated among *A. ruhlandii* isolates than *A. insuavis* or *A. xylosoxidans* isolates. This phenomenon might signal about the continuous adaptive evolution even in highly antibiotic-resistant strains such as DES [1]. Nonetheless, more antibiotic resistance and virulence genes among frequently mutated genes might be identified if gene annotation of *Achromobacter* spp. reference genomes improved. Overall, our identified candidate pathoadaptive genes (belonging to signal transduction; inorganic ion transport and metabolism; intracellular trafficking; transcription; and replication gene functional classes) are comparable to the observations in *P. aeruginosa* infecting patients with CF [50] and other smaller-scale studies on *Achromobacter* spp. [49]

Furthermore, we highlight that not all *Achromobacter* spp. lineages seem to undergo the same amount of selective pressure, and we show from the seven pathoadaptive *Achromobacter* spp. gene analysis that lineages tended to either have acquired not more than two pathoadaptive mutations and be under stronger positive selection or have acquired mutations in more pathoadaptive genes and be under weaker/neutral selection. These findings are comparable to findings from genetic adaptation studies in *P. aeruginosa* from patients with CF airway [50]. Overall, significantly higher d*N*/d*S* ratio in the 27, 16 and 28 most frequently mutated *A. ruhlandii*, *A. insuavis* and *A. xylosoxidans* genes confirms that there is strong selective pressure for changes in these genes during adaptation to the patients with CF airway. Altogether, d*N*/d*S*>1 in both frequently and non-frequently mutated genes show that more than the top 1 % mutated genes are under selective pressure; nonetheless, a larger dataset is needed to identify more genes without sacrificing analysis accuracy.

*AxyZ* (*mexZ* orthologue), which was the only candidate pathoadaptive gene in all three species, is involved in the development of multidrug resistance by regulating AxyXY-OprZ RND-type efflux system, hence is crucial during adaptation to the host environment [37]. *AxyZ* orthologue *mexZ* in *P. aeruginosa* is established among pathoadaptive genes directly associated with increase in antibiotic resistance [51–53]. Furthermore, mutations in *axyZ* could partially explain the increased tolerance to piperacillin/tazobactam and meropenem.

The observed gene loss and acquisition patterns were comparable to the ones observed in *P. aeruginosa*; however, unlike in *P. aeruginosa*, no convergent loss or acquisition of gene clusters was observed [54]

To further explore the differences in antibiotic susceptibility between *Achromobacter* spp. isolates, we performed a k-mer based GWAS analysis. Limited number of bacterial isolates and high innate resistance to certain antibiotics restricted our analysis to only six successful GWAS associations, which revealed that inorganic ion transport genes contribute to antibiotic resistance development in all six antibiotics. Changes in inorganic ion transport genes (COG P) could have a secondary influence on antibiotic resistance as such changes help overcome the problem of iron deficiency in human airways allowing better intrinsically resistant bacteria survival despite the presence of antibiotics. Similar patterns were previously identified in other bacteria causing chronic infections in patients with CF [39, 55, 56]. Another possible explanation is that many inorganic ion transport genes are related to efflux pumps and transporter genes, which markedly contribute to increase in antibiotic resistance [57, 58]. The observed enrichment of transcription genes (COG K) associated with resistance to β-lactams could be explained by changes in transcriptional regulation of intrinsic antibiotic resistance, efflux pump and cell wall protein coding genes [57, 59, 60]. Enrichment of translation and ribosomal structure genes (COG J), and energy production genes (COG C) in trimethoprim/sulfonamide group might be due to altered metabolism and changes in energy production leading to bacterial persistence and escape of antibiotic effect [61, 62]. Similar findings were recently reported by Lopatkin *et al*. [63] where it was shown that mutations in metabolic genes, in particular, central carbon and energy metabolism genes, lead to the increased antibiotic resistance. Ultimately, GWAS is a promising approach for a systematic innately complex bacterial resistance analysis, which could be applied to better understand the genetics of antibiotic resistance development. Gained understanding on how *Achromobacter* spp. develops resistance *in vivo* from our and other studies could be applied to improve the resistance phenotype prediction in the clinic.

Our study has several limitations. First, even larger studies are necessary to further characterize and identify the genetic adaptation of *Achromobacter* spp. during CF airway infections. Second, the lack of genome annotation and overall knowledge about *Achromobacter* spp. limited the interpretation of putative pathoadaptive genes and genes associated with resistance phenotypes. Finally, a single isolate at a given time point is not sufficient to completely reflect the genetic diversity of the bacterial population [64, 65]; therefore, some of our findings might be the result of diversification and not the fixation of the adaptive mutations in *Achromobacter* spp.

In conclusion, by using the largest dataset to date of *Achromobacter* spp. clinical isolates from patients with CF, we used a comprehensive analytical framework for thorough bacterial genomic data analysis. Thus, we identified pathoadaptive and antibiotic resistance genes in *Achromobacter* spp. causing CF airway infections. Furthermore, we showed that current knowledge about antibiotic resistance gene presence or mutations in those genes cannot sufficiently explain the resistance phenotypes, and GWAS offers a new approach of addressing this problem. The gained knowledge allows us to better understand the requirements for successful *Achromobacter* spp. adaptation during infection in airways of patients with CF, which could help accurately predict antibiotic susceptibility and clinical progression of *Achromobacter* spp. infections, and further the development of urgently needed optimized treatment strategies.

## Supplementary Data

Supplementary material 1
