# Supplementary material for: Achromobacter spp. genetic adaptation in cystic fibrosis
Source: Microb Genom. 2021 Jul 7;7(7):000582. doi: 10.1099/mgen.0.000582 (PMC8477396; doi:10.1099/mgen.0.000582)
Supplement: Supplementary material 1 [file mgen-7-0582-s001.pdf]

Table S1. *Achromobacter* spp. lineages, their distribution between species and knowledge about clonal relatedness. DES—Danish epidemic strain.

Species	Lineage	Number of isolates	Patient-to-patient transmission
A. ruhlandii	P1903-AX01DK01	2	Yes (DES) [a]
A. ruhlandii	P3403-AX01DK01	2	Yes (DES) [a]
A. ruhlandii	P4203-AX01DK01	1	Yes (DES) [a]
A. ruhlandii	P4703-AX01DK01	1	Yes (DES) [a]
A. ruhlandii	P5303-AX01DK01	3	Yes (DES) [a]
A. ruhlandii	P7703-AX01DK01	2	Yes (DES) [a]
A. ruhlandii	P8703-AX01DK01	1	Yes (DES) [a]
A. ruhlandii	P9503-AX01DK01	3	Yes (DES) [a]
A. ruhlandii	P9603-AX01DK01	3	Yes (DES) [a]
A. ruhlandii	P3704-AX01DK01	3	Yes (DES) [a]
A. ruhlandii	P5604-AX01DK01	2	Yes (DES) [a]
A. ruhlandii	P6004-AX01DK01	1	Yes (DES) [a]
A. ruhlandii	P1705-AX01DK01	3	Yes (DES) [a]
A. ruhlandii	P7402-AX01DK02	1	No
A. ruhlandii	P4104-AX01DK03	1	No
A. insuavis	P9704-AX02DK01	1	No
A. insuavis	P8504-AX02DK02	2	No
A. insuavis	P9203-AX02DK03	1	No
A. insuavis	P2005-AX02DK04	1	No
A. insuavis	P6004-AX02DK05	1	Potentially [b]
A. insuavis	P6604-AX02DK05	1	Potentially [b]
A. insuavis	P9902-AX02DK06	3	No
A. insuavis	P0802-AX02DK07	2	Potentially [b]
A. insuavis	P0603-AX02DK07	2	Potentially [b]
A. insuavis	P6203-AX02DK07	1	Potentially [b]
A. insuavis	P6203-AX02DK08	1	No
A. insuavis	P5103-AX02DK09	1	No
A. insuavis	P8104-AX02DK10	1	No
A. xylosoxidans	P1700-AX03DK01	1	No
A. xylosoxidans	P6402-AX03DK02	1	No
A. xylosoxidans	P0703-AX03DK02	1	No
A. xylosoxidans	P5204-AX03DK03	1	No
A. xylosoxidans	P8605-AX03DK04	2	No
A. xylosoxidans	P0703-AX03DK05	1	No

A. xylosoxidans	P0803-AX03DK06	3	No
A. xylosoxidans	P0903-AX03DK07	1	No
A. xylosoxidans	P1703-AX03DK08	1	No
A. xylosoxidans	P1903-AX03DK09	2	No
A. xylosoxidans	P2103-AX03DK10	1	No
A. xylosoxidans	P3203-AX03DK11	4	No
A. xylosoxidans	P4203-AX03DK12	1	No
A. xylosoxidans	P4703-AX03DK13	2	No
A. xylosoxidans	P5803-AX03DK14	1	No
A. xylosoxidans	P6203-AX03DK15	1	Potentially [b]
A. xylosoxidans	P6604-AX03DK15	1	Potentially [b]
A. xylosoxidans	P6804-AX03DK15	1	Potentially [b]
A. xylosoxidans	P6203-AX03DK16	1	Potentially [b]
A. xylosoxidans	P7603-AX03DK16	3	Potentially [b]
A. xylosoxidans	P8603-AX03DK17	2	No
A. xylosoxidans	P9203-AX03DK18	2	No
A. xylosoxidans	P9403-AX03DK19	3	No
A. xylosoxidans	P0604-AX03DK20	1	No
A. xylosoxidans	P1704-AX03DK21	2	No
A. xylosoxidans	P3004-AX03DK22	1	No
A. xylosoxidans	P3504-AX03DK23	2	No
A. xylosoxidans	P8404-AX03DK24	3	No
A. xylosoxidans	P9704-AX03DK25	1	No
A. xylosoxidans	P2605-AX03DK26	1	No
A. xylosoxidans	P0610-AX03DK27	2	No
A. xylosoxidans	P5611-AX03DK28	1	No
A. xylosoxidans	P8511-AX03DK29	1	No
Achromobacter sp.	P2202-AX04DK01	1	No
A. aegrifaciens	P7603-AX04DK02	1	No

[a] Ridderberg et al., 2020 [1]; [b] Gabrielaite *et al.*, 2021 [2].

Table S2. Table of publicly available RefSeq *Achromobacter* spp. sequences with original and new proposed annotations which were based on average nucleotide identity (ANI) and core genome SNP-based phylogeny.

**RefSeq ID**	**Original species annotation**	**ANI- and phylogeny-based species annotation**
GCF_001558755.2	*Achromobacter xylosoxidans*	*Achromobacter insuavis*
GCF_001514355.1	*Achromobacter denitrificans*	*Achromobacter ruhlandii*
GCF_001298855.1	*Achromobacter sp.*	*Achromobacter ruhlandii*
GCF_001299395.1	*Achromobacter sp.*	*Achromobacter ruhlandii*
GCF_001051055.1	*Achromobacter xylosoxidans*	*Achromobacter ruhlandii*

GCF_001558915.1	*Achromobacter xylosoxidans*	*Achromobacter ruhlandii*
GCF_001707475.1	*Achromobacter xylosoxidans*	*Achromobacter ruhlandii*
GCF_001707485.1	*Achromobacter xylosoxidans*	*Achromobacter ruhlandii*
GCF_003184165.1	*Achromobacter sp.*	*Achromobacter xylosoxidans*
GCF_001298835.1	*Achromobacter sp.*	*Achromobacter ruhlandii*
GCF_001299135.1	*Achromobacter xylosoxidans*	*Achromobacter dolens*
GCF_001299355.1	*Achromobacter sp.*	*Achromobacter aegrifaciens*
GCF_001428165.1	*Achromobacter sp.*	*Achromobacter mucicolens*
GCF_001975485.1	*Achromobacter sp.*	*Achromobacter mucicolens*
GCF_003267925.1	*Achromobacter sp.*	*Achromobacter pulmonis*
GCF_003576205.1	*Achromobacter sp.*	*Achromobacter aegrifaciens*
GCF_006715415.1	*Achromobacter sp.*	*Achromobacter mucicolens*
GCF_000165835.1	*Achromobacter xylosoxidans*	*Achromobacter sp.*
GCF_001566985.1	*Achromobacter xylosoxidans*	*Achromobacter mucicolens*
GCF_001641525.1	*Achromobacter xylosoxidans*	*Achromobacter mucicolens*
GCF_001654705.1	*Achromobacter xylosoxidans*	*Achromobacter dolens*
GCF_003503255.1	*Achromobacter xylosoxidans*	*Achromobacter sp.*
GCF_002192695.1	*Achromobacter xylosoxidans*	*Achromobacter sp.*
GCF_002192705.1	*Achromobacter xylosoxidans*	*Achromobacter sp.*
GCF_002812705.1	*Achromobacter spanius*	*Achromobacter spanius*
GCF_900636675.1	*Achromobacter spanius*	*Achromobacter spanius*
GCF_900108985.1	*Achromobacter sp.*	*Achromobacter sp.*
GCF_900156745.1	*Achromobacter sp.*	*Achromobacter sp.*
GCF_001189595.1	*Achromobacter sp.*	*Achromobacter sp.*
GCF_003994415.1	*Achromobacter spanius*	*Achromobacter sp.*
GCF_001005835.2	*Achromobacter sp.*	*Achromobacter sp.*
GCF_002926155.1	*Achromobacter spanius*	*Achromobacter sp.*
GCF_003600685.1	*Achromobacter sp.*	*Achromobacter sp.*
GCF_001427425.1	*Achromobacter sp.*	*Achromobacter sp.*
GCF_002966795.1	*Achromobacter spanius*	*Achromobacter sp.*
GCF_002209485.1	*Achromobacter sp.*	*Achromobacter sp.*
GCF_002209495.1	*Achromobacter sp.*	*Achromobacter sp.*
GCF_001298975.1	*Achromobacter sp.*	*Achromobacter insuavis*
GCF_001299435.1	*Achromobacter sp.*	*Achromobacter insuavis*
GCF_001298995.1	*Achromobacter sp.*	*Achromobacter insuavis*
GCF_001299375.1	*Achromobacter sp.*	*Achromobacter insuavis*
GCF_003096315.1	*Achromobacter insuavis*	*Achromobacter insuavis*
GCF_000219745.1	*Achromobacter insuavis*	*Achromobacter insuavis*
GCF_001637085.1	*Achromobacter ruhlandii*	*Achromobacter ruhlandii*
GCF_001637095.1	*Achromobacter ruhlandii*	*Achromobacter ruhlandii*

GCF_001637105.1	*Achromobacter ruhlandii*	*Achromobacter ruhlandii*
GCF_001637115.1	*Achromobacter ruhlandii*	*Achromobacter ruhlandii*
GCF_002082135.1	*Achromobacter ruhlandii*	*Achromobacter ruhlandii*
GCF_002799605.1	*Achromobacter ruhlandii*	*Achromobacter ruhlandii*
GCF_002803005.1	*Achromobacter ruhlandii*	*Achromobacter ruhlandii*
GCF_000517225.1	*Achromobacter xylosoxidans*	*Achromobacter xylosoxidans*
GCF_000967095.2	*Achromobacter xylosoxidans*	*Achromobacter xylosoxidans*
GCF_001038045.1	*Achromobacter xylosoxidans*	*Achromobacter xylosoxidans*
GCF_001057595.1	*Achromobacter xylosoxidans*	*Achromobacter xylosoxidans*
GCF_001057605.1	*Achromobacter xylosoxidans*	*Achromobacter xylosoxidans*
GCF_001275845.1	*Achromobacter xylosoxidans*	*Achromobacter xylosoxidans*
GCF_001275865.1	*Achromobacter xylosoxidans*	*Achromobacter xylosoxidans*
GCF_001275875.1	*Achromobacter xylosoxidans*	*Achromobacter xylosoxidans*
GCF_001276005.1	*Achromobacter xylosoxidans*	*Achromobacter xylosoxidans*
GCF_001276025.1	*Achromobacter xylosoxidans*	*Achromobacter xylosoxidans*
GCF_001276035.1	*Achromobacter xylosoxidans*	*Achromobacter xylosoxidans*
GCF_001276315.1	*Achromobacter xylosoxidans*	*Achromobacter xylosoxidans*
GCF_001298795.1	*Achromobacter xylosoxidans*	*Achromobacter xylosoxidans*
GCF_001298895.1	*Achromobacter xylosoxidans*	*Achromobacter xylosoxidans*
GCF_001298915.1	*Achromobacter xylosoxidans*	*Achromobacter xylosoxidans*
GCF_001298935.1	*Achromobacter xylosoxidans*	*Achromobacter xylosoxidans*
GCF_001299015.1	*Achromobacter xylosoxidans*	*Achromobacter xylosoxidans*
GCF_001299035.1	*Achromobacter xylosoxidans*	*Achromobacter xylosoxidans*
GCF_001299055.1	*Achromobacter xylosoxidans*	*Achromobacter xylosoxidans*
GCF_001299095.1	*Achromobacter xylosoxidans*	*Achromobacter xylosoxidans*
GCF_001299115.1	*Achromobacter xylosoxidans*	*Achromobacter xylosoxidans*
GCF_001299155.1	*Achromobacter xylosoxidans*	*Achromobacter xylosoxidans*
GCF_001299175.1	*Achromobacter xylosoxidans*	*Achromobacter xylosoxidans*
GCF_001299195.1	*Achromobacter xylosoxidans*	*Achromobacter xylosoxidans*
GCF_001299215.1	*Achromobacter xylosoxidans*	*Achromobacter xylosoxidans*
GCF_001299235.1	*Achromobacter xylosoxidans*	*Achromobacter xylosoxidans*
GCF_001299265.1	*Achromobacter xylosoxidans*	*Achromobacter xylosoxidans*
GCF_001299295.1	*Achromobacter xylosoxidans*	*Achromobacter xylosoxidans*
GCF_001299335.1	*Achromobacter xylosoxidans*	*Achromobacter xylosoxidans*
GCF_001299415.1	*Achromobacter xylosoxidans*	*Achromobacter xylosoxidans*
GCF_001471535.2	*Achromobacter xylosoxidans*	*Achromobacter xylosoxidans*
GCF_001541435.1	*Achromobacter xylosoxidans*	*Achromobacter xylosoxidans*
GCF_001559195.1	*Achromobacter xylosoxidans*	*Achromobacter xylosoxidans*
GCF_001598595.1	*Achromobacter xylosoxidans*	*Achromobacter xylosoxidans*
GCF_001807115.1	*Achromobacter xylosoxidans*	*Achromobacter xylosoxidans*

GCF_001810925.1	*Achromobacter xylosoxidans*	*Achromobacter xylosoxidans*
GCF_001813885.1	*Achromobacter xylosoxidans*	*Achromobacter xylosoxidans*
GCF_001815425.1	*Achromobacter xylosoxidans*	*Achromobacter xylosoxidans*
GCF_001815885.1	*Achromobacter xylosoxidans*	*Achromobacter xylosoxidans*
GCF_001957045.1	*Achromobacter xylosoxidans*	*Achromobacter xylosoxidans*
GCF_001957055.1	*Achromobacter xylosoxidans*	*Achromobacter xylosoxidans*
GCF_001957065.1	*Achromobacter xylosoxidans*	*Achromobacter xylosoxidans*
GCF_002083165.2	*Achromobacter xylosoxidans*	*Achromobacter xylosoxidans*
GCF_003031105.1	*Achromobacter xylosoxidans*	*Achromobacter xylosoxidans*
GCF_003177325.1	*Achromobacter xylosoxidans*	*Achromobacter xylosoxidans*
GCF_003293615.1	*Achromobacter xylosoxidans*	*Achromobacter xylosoxidans*
GCF_008432465.1	*Achromobacter xylosoxidans*	*Achromobacter xylosoxidans*
GCF_008631215.1	*Achromobacter xylosoxidans*	*Achromobacter xylosoxidans*
GCF_900475575.1	*Achromobacter xylosoxidans*	*Achromobacter xylosoxidans*
GCF_902363095.1	*Achromobacter xylosoxidans*	*Achromobacter xylosoxidans*
GCF_000186185.1	*Achromobacter xylosoxidans*	*Achromobacter xylosoxidans*
GCF_001457475.1	*Achromobacter xylosoxidans*	*Achromobacter xylosoxidans*
GCF_003939885.1	*Achromobacter aegrifaciens*	*Achromobacter aegrifaciens*
GCF_900496965.1	*Achromobacter agilis*	*Achromobacter agilis*
GCF_004121055.1	*Achromobacter aloeverae*	*Achromobacter aloeverae*
GCF_000236785.1	*Achromobacter arsenitoxydans*	*Achromobacter arsenitoxydans*
GCF_001571365.1	*Achromobacter denitrificans*	*Achromobacter denitrificans*
GCF_001945385.1	*Achromobacter denitrificans*	*Achromobacter denitrificans*
GCF_002192685.1	*Achromobacter denitrificans*	*Achromobacter denitrificans*
GCF_002205315.1	*Achromobacter denitrificans*	*Achromobacter denitrificans*
GCF_003812265.1	*Achromobacter denitrificans*	*Achromobacter denitrificans*
GCF_003939895.1	*Achromobacter denitrificans*	*Achromobacter denitrificans*
GCF_900444675.1	*Achromobacter denitrificans*	*Achromobacter denitrificans*
GCF_003096295.1	*Achromobacter dolens*	*Achromobacter dolens*
GCF_007655095.1	*Achromobacter dolens*	*Achromobacter dolens*
GCF_000783435.2	*Achromobacter insolitus*	*Achromobacter insolitus*
GCF_001639685.1	*Achromobacter insolitus*	*Achromobacter insolitus*
GCF_001641515.1	*Achromobacter insolitus*	*Achromobacter insolitus*
GCF_001707465.1	*Achromobacter insolitus*	*Achromobacter insolitus*
GCF_001971645.1	*Achromobacter insolitus*	*Achromobacter insolitus*
GCF_002209555.1	*Achromobacter insolitus*	*Achromobacter insolitus*
GCF_003293535.1	*Achromobacter insolitus*	*Achromobacter insolitus*
GCF_008245125.1	*Achromobacter insolitus*	*Achromobacter insolitus*
GCF_900637265.1	*Achromobacter insolitus*	*Achromobacter insolitus*
GCF_000286415.1	*Achromobacter marplatensis*	*Achromobacter marplatensis*

GCF_002209535.1	*Achromobacter marplatensis*	*Achromobacter marplatensis*
GCF_003315095.1	*Achromobacter marplatensis*	*Achromobacter marplatensis*
GCF_003053425.1	*Achromobacter mucicolens*	*Achromobacter mucicolens*
GCF_000164035.1	*Achromobacter piechaudii*	*Achromobacter piechaudii*
GCF_001262515.1	*Achromobacter piechaudii*	*Achromobacter piechaudii*
GCF_001571245.1	*Achromobacter piechaudii*	*Achromobacter piechaudii*
GCF_002885955.2	*Achromobacter pulmonis*	*Achromobacter pulmonis*
GCF_000633435.1	*Achromobacter sp.*	*Achromobacter sp.*
GCF_000757485.1	*Achromobacter sp.*	*Achromobacter sp.*
GCF_001270295.1	*Achromobacter sp.*	*Achromobacter sp.*
GCF_001298955.1	*Achromobacter sp.*	*Achromobacter sp.*
GCF_001299315.1	*Achromobacter sp.*	*Achromobacter sp.*
GCF_001428845.1	*Achromobacter sp.*	*Achromobacter sp.*
GCF_002902905.1	*Achromobacter sp.*	*Achromobacter sp.*
GCF_002975275.1	*Achromobacter sp.*	*Achromobacter sp.*
GCF_900496975.1	*Achromobacter sp.*	*Achromobacter sp.*

Table S3. List of most frequently mutated genes in *Achromobacter ruhlandii*, *Achromobacter insuavis* and *Achromobacter xylosoxidans* with their Clusters of Orthologous Groups (COG) function, corresponding E-value, gene description and gene relatedness to antibiotic resistance or virulence with references defining gene's relatedness to antibiotic resistance or virulence are provided where relevant.

**Species**	**E-value (COG function)**	**COG function**	**Number of times the gene was mutated**	**Gene**	**Gene description**	**Virulence gene (ABRicate)**	**Antibiotic resistance gene (ABRicate)**	**Reference**
A. ruhlandii	0	P	5	Axylo_RS04995	magnesium-translocating P-type ATPase	No	Yes	https://jb.asm.org/content/190/14/4951.short
A. ruhlandii	0	T	5	Axylo_RS05365	sensor histidine kinase KdpD	Yes	No	https://journals.plos.org/plospathogens/article?id=10.1371/journal.ppat.1003201;
A. ruhlandii	1.70E-233	E	5	Axylo_RS05465	amino acid deaminase	No	No	
A. ruhlandii	5.70E-113	K	5	Axylo_RS06040	DNA-binding transcriptional repressor AxyZ	No	Yes	https://aac.asm.org/content/61/8/e00290-17.short
A. ruhlandii	0	M	5	Axylo_RS07110	cyanophycin synthetase	Yes	No	https://iai.asm.org/content/81/8/2800.short

A. ruhlandii	0	L	5	Axylo_RS07320	DNA gyrase subunit A	No	Yes	https://academic.oup.com/femsle/article/143/1/25/663481
A. ruhlandii	3.30E-154	K	5	Axylo_RS08030	LysR family transcriptional regulator MetR	Yes	No	https://mbio.asm.org/content/3/5/e00236-12.short
A. ruhlandii	4.70E-159	U	5	Axylo_RS08655	MotA/TolQ/ExbB proton channel family protein	Yes	No	https://www.nature.com/articles/s41598-017-01551-4
A. ruhlandii	3.30E-240	T	5	Axylo_RS09825	HAMP domain-containing protein	No	No	
A. ruhlandii	0	S	5	Axylo_RS11620	Inner membrane component of tripartite multidrug resistance system	No	Yes	https://www.ncbi.nlm.nih.gov/pmc/articles/PMC4543513/
A. ruhlandii	1.70E-256	M	5	Axylo_RS11635	efflux transporter, outer membrane factor (OMF) lipoprotein, NodT family	No	Yes	https://journals.plos.org/plosone/article?id=10.1371/journal.pone.0068484
A. ruhlandii	1.40E-243	T	5	Axylo_RS14635	two-component sensor histidine kinase	No	No	
A. ruhlandii	0	P	5	Axylo_RS17180	multidrug efflux RND transporter permease	No	Yes	https://www.ncbi.nlm.nih.gov/Structure/sparcle/archview.html?archid=11489960
A. ruhlandii	6.40E-257	T	5	Axylo_RS17980	sensor domain-containing diguanylate cyclase	No	No	
A. ruhlandii	1.80E-180	G	5	Axylo_RS19155	DHA2 family efflux MFS transporter permease subunit	No	Yes	https://www.ncbi.nlm.nih.gov/genome/annotation_prok/evidence/TIGR00711/
A. ruhlandii	0	V	5	Axylo_RS23025	ribosome-associated ATPase/putative transporter RbbA	No	Yes	https://www.ncbi.nlm.nih.gov/pmc/articles/PMC3083400/
A. ruhlandii	0	S	5	Axylo_RS23830	WG repeat-containing protein	No	No	
A. ruhlandii	2.60E-285	M	6	Axylo_RS03970	penicillin-binding protein 2	No	Yes	https://www.ncbi.nlm.nih.gov/Structure/sparcle/archview.html?archid=11433609
A. ruhlandii	1.10E-69	V	6	Axylo_RS05925	AXC family carbapenem-hydrolyzing class A beta-lactamase	No	Yes	https://www.ncbi.nlm.nih.gov/genome/annotation_prok/evidence/NF033929/
A. ruhlandii	0	P	6	Axylo_RS06975	TonB-dependent siderophore receptor	Yes	No	https://academic.oup.com/femsre/article/38/4/569/755321
A. ruhlandii	5.40E-199	E	6	Axylo_RS08835	polyamine ABC transporter ATP-binding protein	No	No	
A. ruhlandii	0	T	6	Axylo_RS10450	hybrid sensor histidine kinase/response regulator	No	No	

A. ruhlandii	0	U	6	Axylo_RS12535	filamentous hemagglutinin N-terminal domain-containing protein	Yes	No	https://mbio.asm.org/content/6/4/e01189-15.short
A. ruhlandii	0	P	6	Axylo_RS21160	TonB-dependent hemin, ferrichrome receptor	Yes	No	https://www.mdpi.com/2073-4425/7/12/126/htm
A. ruhlandii	0	T	6	Axylo_RS22105	response regulator	No	No	
A. ruhlandii	0	U	5	Axylo_RS25190	ShlB/FhaC/HecB family hemolysin secretion/activation protein	Yes	No	https://www.tandfonline.com/doi/full/10.1080/21505594.2016.1262313
A. ruhlandii	-	-	7	Axylo_RS26755	pseudogene	No	No	
A. insuavis	5.80E-113	K	2	AL504_RS00240	MarR family transcriptional regulator	No	Yes	https://www.ncbi.nlm.nih.gov/Structure/sparcle/archview.html?archid=12204203
A. insuavis	2.70E-115	P	2	AL504_RS02460	taurine import ATP-binding protein TauB	No	No	
A. insuavis	0	P	2	AL504_RS03095	chloride channel protein	No	No	
A. insuavis	2.80E-280	E	2	AL504_RS12045	leucyl aminopeptidase	No	No	
A. insuavis	2.20E-194	D	2	AL504_RS12415	membrane protein	No	No	
A. insuavis	1.40E-114	K	2	AL504_RS12625	DNA-binding transcriptional repressor AxyZ	No	Yes	https://aac.asm.org/content/61/8/e00290-17.short
A. insuavis	1.80E-224	P	2	AL504_RS15290	hypothetical protein	No	No	
A. insuavis	-	-	2	AL504_RS15295	hypothetical protein	No	No	
A. insuavis	0	L	2	AL504_RS15960	DUF342 domain-containing protein	No	No	
A. insuavis	8.20E-166	O	2	AL504_RS17075	NAD(P)/FAD-dependent oxidoreductase	No	No	
A. insuavis	0	J	2	AL504_RS19685	elongation factor G	No	Yes	https://onlinelibrary.wiley.com/doi/epdf/10.1002/pmic.200500112
A. insuavis	1.50E-253	J	2	AL504_RS21310	16S rRNA (cytosine(967)-C(5))-methyltransferase RsmB	No	No	
A. insuavis	3.00E-266	G	2	AL504_RS22030	MFS transporter	No	Yes	https://www.ncbi.nlm.nih.gov/Structure/sparcle/archview.html?archid=13022956
A. insuavis	0	E	2	AL504_RS22980	lysine decarboxylase	Yes	No	https://www.sciencedirect.com/science/article/abs/pii/S0969212619303442
A. insuavis	0	L	2	AL504_RS27775	DNA mismatch repair protein MutS	No	No	
A. insuavis	0	M,U	3	AL504_RS04195	hypothetical protein	Yes	No	https://www.sciencedirect.com/science/article/abs/pii/S1438422100801022

A. xylosoxidans	4.10E-97	-	3	AT699_RS01280	hypothetical protein	No	No	
A. xylosoxidans	4.00E-165	O	3	AT699_RS01870	NAD(P)/FAD-dependent oxidoreductase	No	No	
A. xylosoxidans	6.40E-156	S	3	AT699_RS05145	tripartite tricarboxylate transporter substrate binding protein BugD	No	No	
A. xylosoxidans	2.50E-108	M	3	AT699_RS06160	outer membrane lipoprotein chaperone LolA	No	No	
A. xylosoxidans	1.10E-111	K	3	AT699_RS06735	DNA-binding transcriptional repressor AxyZ	No	Yes	https://aac.asm.org/content/61/8/e00290-17.short
A. xylosoxidans	5.70E-210	I	3	AT699_RS07205	beta-ketoacyl-ACP synthase II	No	No	
A. xylosoxidans	0	P	3	AT699_RS11865	multidrug efflux RND transporter permease	No	Yes	https://www.ncbi.nlm.nih.gov/Structure/sparcle/archview.html?archid=11489960
A. xylosoxidans	0	S	3	AT699_RS12875	AAA family ATPase	No	No	
A. xylosoxidans	0	F	3	AT699_RS13295	bifunctional (p)ppGpp synthetase/guanosine-3',5'-bis(diphosphate) 3'-pyrophosphohydrolase	No	No	
A. xylosoxidans	4.00E-260	Q	3	AT699_RS14700	SidA/IucD/PvdA family monooxygenase	Yes	No	https://onlinelibrary.wiley.com/doi/full/10.1002/mbo3.804
A. xylosoxidans	4.00E-90	T	3	AT699_RS18500	low molecular weight phosphotyrosine protein phosphatase	No	No	
A. xylosoxidans	2.40E-151	K	3	AT699_RS21090	IclR family transcriptional regulator	Yes	No	https://www.ncbi.nlm.nih.gov/Structure/sparcle/archview.html?archid=11444403
A. xylosoxidans	2.40E-238	T	3	AT699_RS22580	HAMP domain-containing protein	No	No	
A. xylosoxidans	0	P	3	AT699_RS25090	TonB-dependent hemin, ferrichrome receptor	Yes	No	https://www.mdpi.com/2073-4425/7/12/126/htm
A. xylosoxidans	2.00E-164	K	3	AT699_RS29085	LysR family transcriptional regulator	Yes	No	https://www.ncbi.nlm.nih.gov/Structure/sparcle/archview.html?archid=10444056
A. xylosoxidans	7.80E-53	K	3	AT699_RS29425	helix-turn-helix transcriptional regulator	No	No	
A. xylosoxidans	7.40E-162	E	3	AT699_RS30730	pyridoxal-phosphate dependent enzyme	No	No	
A. xylosoxidans	9.00E-215	P	3	AT699_RS30735	catalase	No	No	
A. xylosoxidans	0	L	4	AT699_RS00345	DNA topoisomerase III	No	Yes	https://academic.oup.com/femspd/article/67/3/159/2398791

A. xylosoxidans	3.60E-287	M	4	AT699_RS03990	penicillin-binding protein 2	No	Yes	https://www.ncbi.nlm.nih.gov/Structure/sparcle/archview.html?archid=11433609
A. xylosoxidans	2.00E-167	K	4	AT699_RS11855	LysR family transcriptional regulator	Yes	No	https://onlinelibrary.wiley.com/doi/full/10.1111/j.1365-2958.2006.05269.x
A. xylosoxidans	1.10E-263	T	4	AT699_RS15300	HAMP domain-containing histidine kinase	Yes	No	https://www.embopress.org/doi/full/10.1093/emboj/cdg159
A. xylosoxidans	1.20E-95	T	4	AT699_RS25225	response regulator transcription factor	No	No	
A. xylosoxidans	3.60E-234	T	4	AT699_RS26600	HAMP domain-containing histidine kinase	Yes	No	https://www.embopress.org/doi/full/10.1093/emboj/cdg159
A. xylosoxidans	0	S	4	AT699_RS26820	hypothetical protein	No	No	
A. xylosoxidans	6.90E-205	S	5	AT699_RS03090	LPS export ABC transporter permease LptG	No	Yes	https://onlinelibrary.wiley.com/doi/full/10.1002/mbo3.844
A. xylosoxidans	2.10E-209	P	6	AT699_RS03490	ABC transporter substrate-binding protein	No	No	

Table S4. All mutations acquired by lineages in the 7 pathoadaptive genes which were frequently mutated in at least two *Achromobacter* species. Amino acid coordinates are based on the corresponding species' reference genome genes.

**Lineage**	**AT699_RS06735/ Axylo_RS06040/ AL504_RS12625**	**AT699_RS22580/ Axylo_RS09825/ AL504_RS27430**	**AT699_RS11865/ Axylo_RS17180/ AL504_RS06700**	**AT699_RS03990/ Axylo_RS03970/ AL504_RS14780**	**AT699_RS25090/ Axylo_RS21160/ AL504_RS25170**	**AT699_RS03490/ Axylo_RS03480/ AL504_RS15290**	**AT699_RS01870/ Axylo_RS01775/ AL504_RS17075**
P1903-AX01DK01			c.2041G>A, p.Asp681Asn; c.904A>G, p.Thr302Ala; c.472G>A, p.Val158Ile	c.623T>C, p.Val208Ala	c.213C>T, p.Asn71Asn		
P3403-AX01DK01	c.7C>T,; p.Arg3Cys	c.957G>A, p.Thr319Thr; c.857G>A, p.Arg286His			c.2531C>G, p.Thr844Arg; c.1574A>G, p.His525Arg	c.307T>C, p.Tyr103His	
P5303-AX01DK01		c.841G>A, p.Gly281Ser	c.1106C>T, p.Thr369Met	c.937G>A, p.Gly313Ser	c.1531A>G, p.Thr511Ala		
P7703-AX01DK01							
P9503-AX01DK01		c.565G>A, p.Ala189Thr	c.188A>G, p.Gln63Arg	c.1372A>G, p.Thr458Ala; c.1591G>A,	c.1141A>C, p.Ser381Arg;		

				p.Gly531Ser; c.1594G>A, p.Gly532Ser	c.1117A>G, p.Met373Val		
P9603-AX01DK01	c.139G>A, p.Ala47Thr	c.745A>G, p.Thr249Ala; c.101C>T, p.Ser34Leu	c.2324A>G, p.Glu775Gly; c.188A>G, p.Gln63Arg	c.1040C>T, p.Ala347Val; c.1391T>C, p.Met464Thr	c.2531C>G, p.Thr844Arg		
P3704-AX01DK01	c.488A>G, p.Tyr163Cys			c.1507C>T, p.Arg503Trp; c.1594G>A, p.Gly532Ser			
P5604-AX01DK01	c.76T>C, p.Ser26Pro; c.187T>C, p.Cys63Arg	c.923T>C, p.Val308Ala	c.838T>C, p.Tyr280His	c.274C>T, p.Pro92Ser; c.740A>G, p.His247Arg			
P1705-AX01DK01	c.149G>A, p.Gly50Asp		c.323A>G, p.Gln108Arg		c.1232dupG, p.Trp412fs		
P0802-AX02DK07	c.496T>C, p.Ser166Pro; c.35C>T, p.Thr12Ile				c.1547A>G, p.His516Arg	c.581T>C, p.Leu194Pro	
P9902-AX02DK06	c.146A>G, p.Tyr49Cys		c.3002T>C, p.Ile1001Thr			c.871C>A, p.Arg291Ser	c.127A>C, p.Ser43Arg
P0603-AX02DK07							c.619C>T, p.Gln207*
P8504-AX02DK02							
P0803-AX03DK06							.
P1903-AX03DK09						c.78_80dupGGC, p.Ala27dup	
P3203-AX03DK11		c.1120G>A, p.Gly374Ser	c.173A>G, p.Gln58Arg; c.799C>T, p.Arg267Cys; c.2033A>G, p.Asp678Gly; c.2480A>G, p.Gln827Arg; c.2891A>G, p.His964Arg	c.1069G>A, p.Gly357Ser; c.1208T>C, p.Leu403Pro; c.1283T>C, p.Val428Ala; c.1420A>G, p.Thr474Ala; c.1508G>A, p.Arg503His; c.1557C>T, p.Ile519Ile	c.1141A>C, p.Ser381Arg	c.78_80dupGGC, p.Ala27dup	c.26dupG, p.Gly10fs; c.440A>G, p.Tyr147Cys; c.473dupG, p.Asp159fs
P4703-AX03DK13		c.633A>C, p.Glu211Asp		c.1430C>A, p.Ala477Asp			
P7603-AX03DK16	c.462_463delGC, p.Leu155fs				c.370C>T, p.Pro124Ser	c.578G>A, p.Ser193Asn; c.382T>G, p.Phe128Val	c.26dupG, p.Gly10fs
P8603-AX03DK17							

P9203-AX03DK18	c.462_463delGC, p.Leu155fs					c.78_80dupGGC, p.Ala27dup	
P9403-AX03DK19	c.508G>A, p.Gly170Ser		c.490G>A, p.Asp164Asn	c.1594G>A, p.Gly532Ser	c.1143C>G, p.Ser381Arg		
P1704-AX03DK21							
P3504-AX03DK23							
P8404-AX03DK24						c.78_80dupGGC, p.Ala27dup	
P8605-AX03DK04						c.78_80dupGGC, p.Ala27dup	
P0610-AX03DK27		c.125G>A, p.Arg42His	c.266C>T, p.Ser89Phe	c.218A>G, p.Asp73Gly; c.1031T>G, p.Leu344Arg			c.26dupG, p.Gly10fs

Table S5. Table of pan-genome, core, accessory and unique genes in *Achromobacter* spp. lineages. Variable genes are separated by whether they were (1) lost or acquired and (2) variable in a group or individually.

**Lineage**	**Pan-genome size**	**Core genome**	**Accessory genome**	**Unique genes**	**Lost genes**	**Acquired genes**	**Lost/acquired in group**	**Lost/acquired individually**
P0802-AX02DK07	6299	6284	15	15	12	3	14	1
P9902-AX02DK06	6031	5964	67	26	60	7	61	6
P0603-AX02DK07	6154	6081	73	73	24	49	71	2
P0803-AX03DK06	6377	6240	137	95	42	95	137	0
P1903-AX01DK01	5751	5717	34	34	0	34	34	0
P1903-AX03DK09	6315	6230	85	85	83	2	84	1
P3203-AX03DK11	5733	5642	91	30	60	31	89	2
P3403-AX01DK01	5709	5684	25	25	24	1	23	2
P4703-AX03DK13	5754	5587	167	167	156	11	167	0
P5303-AX01DK01	5732	5698	34	34	33	1	33	1
P7603-AX03DK16	5981	5953	28	22	10	18	28	0
P7703-AX01DK01	5740	5708	32	32	16	16	30	2
P8603-AX03DK17	6065	6004	61	61	52	9	61	0
P9203-AX03DK18	5762	5738	24	24	16	8	23	1
P9403-AX03DK19	5981	5945	36	33	6	30	35	1
P9503-AX01DK01	5855	5700	155	122	100	55	153	2
P9603-AX01DK01	5709	5695	14	12	6	8	13	1
P1704-AX03DK21	5850	5812	38	38	36	2	34	4
P3504-AX03DK23	6139	6109	30	30	14	16	30	0
P3704-AX01DK01	5907	5724	183	128	87	96	181	2
P5604-AX01DK01	5750	5725	25	25	23	2	23	2

P8404-AX03DK24	6230	6135	95	14	87	8	91	4
P8504-AX02DK02	6129	6108	21	21	NA	NA	20	1
P1705-AX01DK01	5749	5714	35	20	4	31	32	3
P8605-AX03DK04	5753	5708	45	44	3	42	42	3
P0610-AX03DK27	5983	5910	73	73	69	4	72	1

Table S6. The most significant genome-wide association study (GWAS) associations (q-value<0.05) and their annotations.

**q-value (comp)**	**Estimated effect**	**Reference genome annotation**	**COG annotation**	**Locus tag (A. xylosoxidans)**	**Locus tag (A. insuavis)**	**Locus tag (A. ruhlandii)**	**Accessory or core genome**
2.44E-03	1.27/1.33	outer membrane lipoprotein LolB	M	AT699_RS26510	AL504_RS23260	Axylo_RS22610	Accessory
2.44E-03	1.33	radical SAM protein	C	AT699_RS17605	AL504_RS00905	Axylo_RS12770	Accessory
3.53E-03	-0.97/1.11	pyruvate, phosphate dikinase	G	AT699_RS11685	AL504_RS06890	Axylo_RS17375	Core
5.99E-03	1.04	ABC transporter substrate-binding protein	P	AT699_RS00340	AL504_RS19000	Axylo_RS00350	Core
8.45E-03	1.65	aminoacyl-tRNA hydrolase	J	AT699_RS12635	AL504_RS05565	Axylo_RS13300	Accessory
1.06E-02	1.04	ABC transporter ATP-binding protein	P	AT699_RS10200	AL504_RS08485	Axylo_RS18875	Core
1.06E-02	1.24	radical SAM protein	C	AT699_RS17605	AL504_RS00905	Axylo_RS12770	Accessory
1.06E-02	1.28	MFS transporter	EGP	AT699_RS21015	AL504_RS28990		Core
1.06E-02	-1.41	TetR/AcrR family transcriptional regulator	K	AT699_RS11830	AL504_RS06735	Axylo_RS17215	Accessory
2.85E-02	-1.05/1.11	DotU family type IV/VI secretion system protein	N	AT699_RS16150	AL504_RS01895		Core
8.45E-06	1.41	TonB-dependent siderophore receptor; amino acid ABC transporter substrate-binding	P	AT699_RS06625		Axylo_RS05930	Accessory
6.60E-05	1.37	Putative hydrolase/carboxylic esterase	S	AT699_RS00630	AL504_RS18635	Axylo_RS00580	Core
6.69E-05	1.37	organic hydroperoxide resistance protein	O	AT699_RS22585	AL504_RS27425	Axylo_RS09820	Core
6.69E-05	1.37	DNA mismatch repair protein MutS	L	AT699_RS22150	AL504_RS27775	Axylo_RS10195	Accessory
6.69E-05	1.37	Transcriptional regulator, AraC family	K	AT699_RS03405	AL504_RS15545	Axylo_RS03205	Core
6.69E-05	1.37	LysR family transcriptional regulator	K	AT699_RS11620[a]	AL504_RS06955[a]	Axylo_RS17440[a]	Core
6.69E-05	-0.94	ATP-dependent RNA helicase	JKL	AT699_RS29520	AL504_RS20735	Axylo_RS25200	Accessory

1.33E-04	1.65	multidrug efflux RND transporter permease AxyY	V	AT699_RS06725	AL504_RS12635	Axylo_RS06030	Core
1.33E-04	1.36	multidrug transporter subunit MdtB	V	AT699_RS02790	AL504_RS16140	Axylo_RS02585	Core
1.33E-04	1.33	Filamentous hemagglutinin; pseudogene	-	AT699_RS29505	AL504_RS11560	Axylo_RS25185	
2.24E-02	1.5	amine dehydrogenase	C	AT699_RS09615	AL504_RS09355	Axylo_RS19475	Core
2.24E-02	1.48	multidrug efflux RND transporter permease AxyY	V	AT699_RS06725	AL504_RS12635	Axylo_RS06030	Core
2.24E-02	1.47	hypothetical protein	P	AT699_RS06895	AL504_RS12460	Axylo_RS06195	Accessory
2.24E-02	1.47	Acyl-CoA dehydrogenase	I	Uniprot ID: A0A1D8I881	Uniprot ID: A0A1D8I881	Uniprot ID: A0A1D8I881	Accessory
2.38E-02	1.39	TonB-dependent siderophore receptor	P	AT699_RS21215	AL504_RS28745		Accessory
2.38E-02	1.38	FUSC family protein	S	AT699_RS15550	AL504_RS05585	Axylo_RS13280	Core
2.38E-02	1.39	NADH-quinone oxidoreductase subunit G	C	AT699_RS06820	AL504_RS12540	Axylo_RS06125	Core
2.38E-02	1.40	AraC family transcriptional regulator	K	AT699_RS22495	AL504_RS27515	Axylo_RS09915	Core
2.38E-02	1.39	TonB-dependent siderophore receptor	P	AT699_RS21215	AL504_RS28745		Accessory
2.38E-02	1.39	murein transglycosylase	M	AT699_RS01840	AL504_RS17105	Axylo_RS01745	Core
3.95E-03	1.29	DUF2809 domain-containing protein	S	AT699_RS01515	AL504_RS17430	Axylo_RS01515	Accessory
3.95E-03	1.31	AraC family transcriptional regulator; GlxA family transcriptional regulator	K	AT699_RS19725	AL504_RS30075	Axylo_RS11170	Accessory
3.95E-03	1.33	endolytic transglycosylase MltG	S	AT699_RS12475	AL504_RS02235	Axylo_RS16545	Accessory
3.95E-03	1.13	AraC family transcriptional regulator	K	AT699_RS22495	AL504_RS27515	Axylo_RS09915	Core
3.95E-03	-1.04	branched-chain amino acid ABC transporter permease	U	AT699_RS04800	AL504_RS13950	Axylo_RS04730	Core
4.39E-03	1.22	hypothetical protein	S	AT699_RS00475		Axylo_RS00495	Accessory
4.39E-03	1.22	Putative manganese efflux pump MntP	P		AL504_RS27680	Axylo_RS10100	Accessory
4.39E-03	1.12	N-carbamoylsarcosine amidase	Q	AT699_RS16385	AL504_RS01610		Core
4.39E-03	1.16	type I secretion system permease/ATPase	P	AT699_RS27360	AL504_RS22385	Axylo_RS23340	Accessory
4.96E-03	1.13	MarR family transcriptional regulator	K	AT699_RS26215	AL504_RS23535	Axylo_RS22350	Accessory
6.66E-03	1.38	16S rRNA	J	AT699_RS04335	AL504_RS14405	Axylo_RS04330	Core

6.66E-03	1.38	protein-disulfide reductase DsbD	CO	AT699_RS30205	AL504_RS20050	Axylo_RS25815	Core
6.66E-03	1.38	iron ABC transporter permease	P	AT699_RS13445	AL504_RS04765	Axylo_RS14080	Core
9.89E-03	1.38	TonB-dependent siderophore receptor	P	AT699_RS21215	AL504_RS28745		Accessory
9.89E-03	1.38	FUSC family protein	S		AL504_RS30540	Axylo_RS11620	Accessory
9.89E-03	1.38	LD-carboxypeptidase	V	AT699_RS14215	AL504_RS03960	Axylo_RS14760	Accessory
9.89E-03	1.38	NADH-quinone oxidoreductase subunit G	C	AT699_RS06820	AL504_RS12540	Axylo_RS06125	Core
9.89E-03	-1.28/1.38	murein transglycosylase	M	AT699_RS01840	AL504_RS17105	Axylo_RS01745	Core
9.89E-03	1.38	imidazolonepropionase	Q	AT699_RS01460	AL504_RS17485	Axylo_RS01460	Accessory
9.89E-03	1.38	Ferrichrome receptor FcuA	P	AT699_RS21215	AL504_RS28745		Accessory
1.32E-02	-1.22/1.49	ABC transporter ATP-binding protein	V	AT699_RS21650	AL504_RS28325	Axylo_RS10590	Core
1.32E-02	-1.23	uracil-DNA glycosylase	L	AT699_RS06925	AL504_RS12430	Axylo_RS06225	Accessory
1.32E-02	1.36	GNAT family N-acetyltransferase	J			Axylo_RS23215	Accessory
1.32E-02	-1.23	VgrG protein; type IV secretion protein Rhs	S	AT699_RS16180		Axylo_RS14380	Accessory
2.51E-02	1.24	16S rRNA	J	AT699_RS04335	AL504_RS14405	Axylo_RS04330	Core
2.51E-02	-1.30	glutamate carboxypeptidase	E	AT699_RS13095	AL504_RS05100	Axylo_RS13750	Accessory
2.51E-02	1.30	endolytic transglycosylase MltG	S	AT699_RS12475	AL504_RS02235	Axylo_RS16545	Accessory
2.51E-02	1.12	TonB-dependent siderophore receptor	P		AL504_RS00915	Axylo_RS12780	Accessory
2.51E-02	-1.21	50S ribosomal protein L3 N(5)-glutamine	J	AT699_RS19080	AL504_RS30750	Axylo_RS11810	Core
2.51E-02	-1.11/-1.18	NADH-quinone oxidoreductase subunit NuoH	C	AT699_RS06825	AL504_RS12535	Axylo_RS06130	Core

[a] Pathoadaptive gene

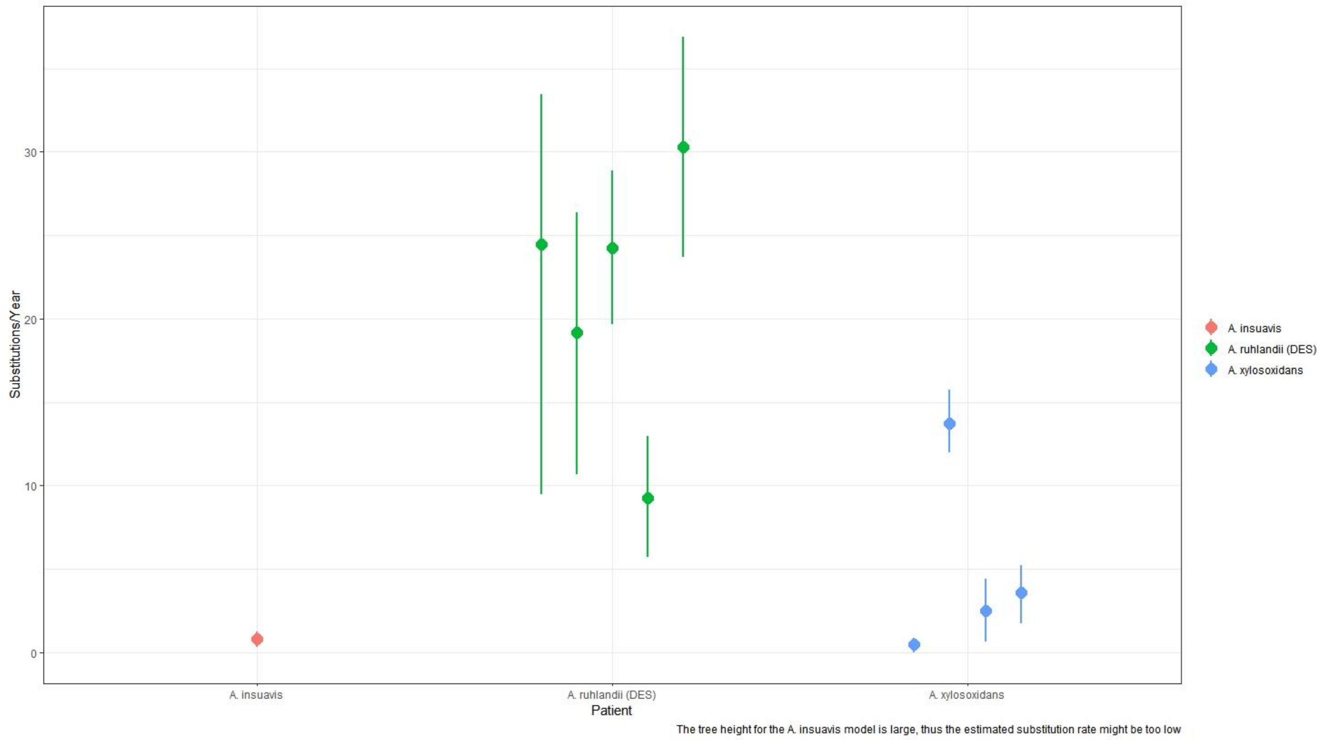

Figure S1. Substitution rate per year average with 95% confidence intervals for *Achromobacter insuavis*, *Achromobacter ruhlandii* (Danish epidemic strain (DES)) and *Achromobacter xylosoxidans*.

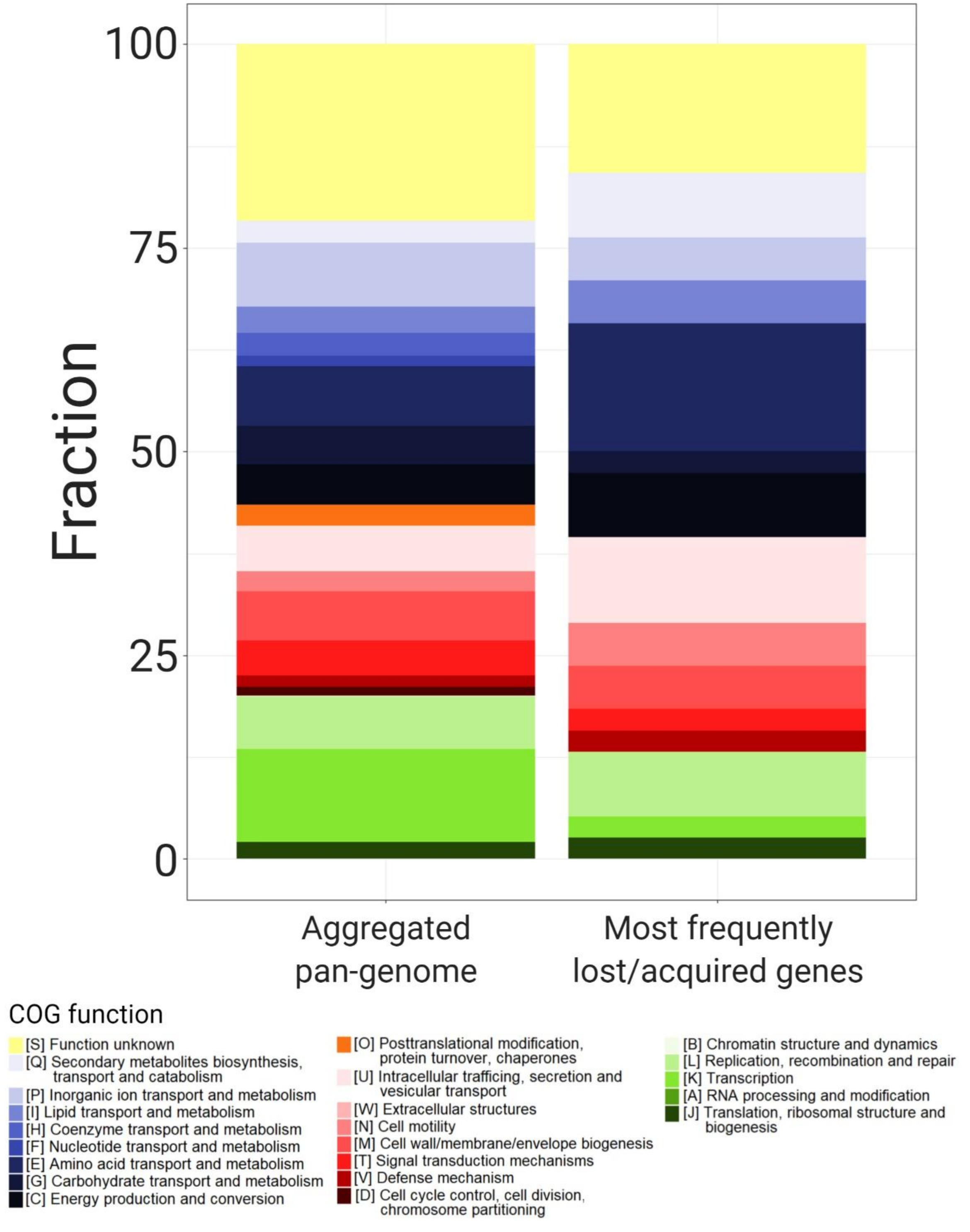

Figure S2. Clusters of Orthologous Groups (COG) annotation of the aggregated pan-genome and most frequently lost or acquired genes.

Text S1. *Achromobacter* spp. gene presence-absence analysis results.

As a crucial part of bacterial adaptation and evolution, we investigated the most frequently lost and acquired genes in the 26 longitudinally sampled *Achromobacter* spp. lineages. First, *de novo* assembled genomes were annotated using Prokka version 1.12 [3] with the settings of a minimum contig length of 200 nucleotides and using a manually created annotation database for *Achromobacter* spp. (GCF_001051055.1 (*A. ruhlandii*), GCF_001558755.2 (*A. insuavis*) and GCF_001457475.1) (*A. xylosoxidans*). Further analysis was based on longitudinal lineages by GenAPI software with default settings. [4] Genes shorter than 150 nucleotides were excluded from further analysis by default. We defined a gene as lost if it was present in the first isolate but absent in one or more of the later isolates and a gene was defined as acquired if it was absent in the first isolate but present in one or more of the later isolates. The lineage pan-genomes contained on average 5,940 (5,709–6,377) genes of which on average 62 (14–183) were lost or acquired. In total, we found 1,464 genes to be variable within lineages and 735 of these were unique to a single lineage in the aggregated pan-genome (Figure 2B). We observed that genes were 2 times more often lost than acquired and lost/acquired in groups 37 times more commonly than individually (Table S5).

Most frequently lost or acquired genes were defined if a gene was lost/acquired in a minimum of 3 lineages. In total, 38 genes passed these criteria; however, after the manual inspection, one gene was defined as a false positive: the gene was manually assessed as present, yet the gene was not assembled with a minimum requirement of 25% of the gene length coverage, thus was predicted to be absent by GenAPI. The remaining 37 genes that were frequently lost or acquired, were annotated with EGGNOG-mapper: transport and metabolism genes were more frequently lost/acquired, especially amino acid transport and metabolism genes, when compared to the composition of the aggregated pan-genome. Contrary, structural, cellular organization and cell cycle genes; reproduction; and gene expression regulating genes were less frequently lost/acquired (Figure S2).

References

[1] Ridderberg W, Jensen Handberg K, Nørskov-Lauritsen N. Prevalence of hypermutator isolates of Achromobacter spp. from cystic fibrosis patients. Int J Med Microbiol. 2020 Jan 16;151393.

[2] Gabrielaite M, Bartell JA, Nørskov-Lauritsen N, Pressler T, Nielsen FC, Johansen HK, et al. Transmission and antibiotic resistance of Achromobacter in cystic fibrosis. J Clin Microbiol. 2021 Jan 20.
[3] Seemann T. Prokka: rapid prokaryotic genome annotation. Bioinformatics. 2014 Jul 15;30(14):2068–9.
[4] Gabrielaite M, Marvig RL. GenAPI: a tool for gene absence-presence identification in fragmented bacterial genome sequences. BMC Bioinformatics. 2020 Jul 20;21(1):320.
